# Photochemical Transformations of Tetrazole Derivatives: Applications in Organic Synthesis

**DOI:** 10.3390/molecules15053757

**Published:** 2010-05-25

**Authors:** Luís Miguel Teodoro Frija, Amin Ismael, Maria Lurdes Santos Cristiano

**Affiliations:** 1 CQFM - Centro de Química-Física Molecular, IN - Institute of Nanoscience and Nanotechnology, Departamento de Engenharia Química e Biológica, Instituto Superior Técnico, Av. Rovisco Pais 1, 1049-001 Lisboa, Portugal; E-Mail: luisfrija@ist.utl.pt (L.M.T.F.); 2 Departmento de Química e Farmácia, FCT, and CCMAR, Universidade do Algarve, Campus de Gambelas, 8005-039 Faro, Portugal

**Keywords:** tetrazoles, photochemistry, UV-irradiation, nitrogen heterocyles, synthesis

## Abstract

Tetrazoles remain a challenge to photochemists. Photolysis leads to cleavage of the tetrazolyl ring, may involve various photodegradation pathways and may produce a diversity of photoproducts, depending on the structure and conformational flexibility of the substituents and the possibility of tautomerism. If the photochemistry of tetrazoles is considered within the frame of synthetic applications the subject is even more challenging, since the ultimate goal is to achieve selectivity and high yield. In addition, the photoproducts must remain stable and allow isolation or trapping, in order to be used in other reactions. This review summarises the photochemical transformations of tetrazole derivatives that can be used as effective synthetic routes to other compounds.

## 1. Introduction

**Figure 1:**
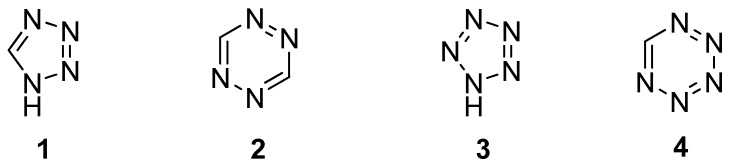
Structures of *1H*-tetrazole (**1**), 1,2,4,5-tetrazine (**2**), *1H*-pentazole (**3**) and pentazine (**4**).

Tetrazole and its derivatives have important applications in major areas, such as medicine, agriculture and imaging technology, and are very stimulating heterocycles from an academic viewpoint. Tetrazole (CN_4_H_2_, **1**, Figure 1) exhibits tautomerism [1], and its nitrogen content is 80% of the total weight of the molecule, the largest percentage among stable unsubstituted heterocyclic systems. In this respect, tetrazole (**1**) surpasses tetrazine (**2**) and is inferior only to some unstable heterocyclic systems practically nonexistent in the free state, such as pentazoles (**3**) and pentazines (**4**) (structures in Figure 1). 

Despite the high nitrogen content, tetrazole and most of its derivatives are relatively stable, on heating or under microwave irradiation and also in the presence of various chemical reagents (oxidants, acids, bases, alkylating agents, dienophiles, *etc.*). In naturally occurring molecules, the tetrazole fragment is virtually lacking. Yet, its presence in metabolic products of some protozoa was reported [2]. It is postulated that tetrazole, alongside other unusual polynitrogen heterocycles, may be formed under the natural conditions of other planets of the Solar system or their satellites, provided that they contain hydrocarbons and nitrogen in the composition of the atmosphere or on the surface [2]. 

The tetrazolyl system is to the same extent unusual in structure and unique in acid-base characteristics. For instance, compared with other thermally and chemically stable azoles, tetrazoles possess abnormally high acidity and very weak basicity [2,3,4,5,6,7,8,9,10,11,12,13]. In the tetrazole ring, the four nitrogen atoms connected in succession may be involved in protolytic processes, and many physical, chemical, physicochemical, and biological properties of tetrazoles are closely related to their ability to behave as acids and bases. In fact, most medical applications of tetrazoles stem from the acidic properties of the tetrazolyl ring. The tetrazolic acid fragment, –CN_4_H, has similar acidity to the carboxylic acid group, –CO_2_H, and is almost allosteric with it, but is metabolically more stable at physiologic pH. Hence, synthetic methodologies leading to the replacement of –CO_2_H groups by –CN_4_H groups in pharmacologically active molecules are of major relevance [14]. The tetrazole ring is found in drugs or drug candidates with antihypertensive, antiallergic and antibiotic activity [15,16,17], or of use as anticonvulsants [18], in cancer or in AIDS treatments [19,20]. Tetrazoles are also used in agriculture as plant growth regulators, herbicides and fungicides [21], or in photography and photoimaging as stabilizers [10]. Due to the high enthalpy of formation, tetrazole decomposition results in the liberation of two nitrogen molecules and a significant amount of energy. Therefore, several tetrazole derivatives have been explored as explosives, propellant components for missiles and as gas generators for air-bags in the automobile industry [22]. In addition, various tetrazole-based compounds have good coordination properties and are able to form stable complexes with several metal ions [23]. This ability is successfully used in analytical chemistry for the removal of heavy metal ions from liquids, and in chemical systems formulated for metal protection against corrosion [24]. Furthermore, the tetrazole ring has strong electron-withdrawing properties and, as such, tetrazolyl halides have been successfully used in synthesis as derivatising agents for the chemical modification of alcohols [25,26,27,28,29,30].

**Scheme 1:**
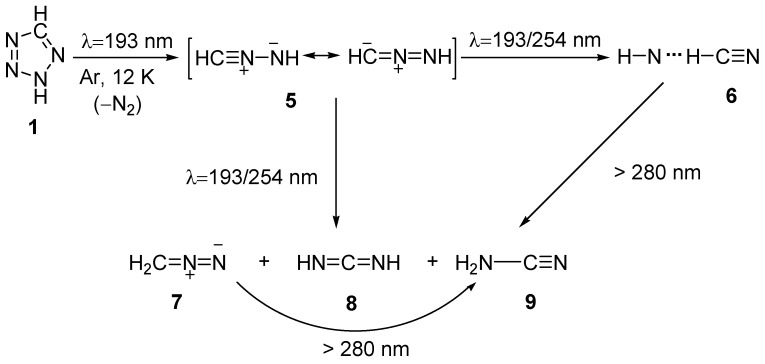
Photodecomposition of matrix-isolated unsubstituted tetrazole.

Tetrazoles are also particularly interesting compounds because they exhibit a very rich photochemistry. The photochemistry of matrix-isolated unsubstituted tetrazole was studied by Maier and co-workers and published in 1996 [31]. Upon photolysis with the 193 nm emission line of an ArF laser, rapid photocleavage of tetrazole was observed, leading to extrusion of N_2_ and formation of several different photoproducts, including nitrilimine (**5**), an HCN···NH complex (**6**), diazomethane (**7**), carbodiimide (**8**) and cyanamide (**9**) (Scheme 1). By use of different excitation wavelengths, cyanamide and carbodiimide could be accumulated as final products. This investigation also revealed, for the first time, the vibrational signature of matrix-isolated nitrilimine [31].

Several tetrazole derivatives have also been studied regarding their photochemical fragmentation reactions, matrix-isolated or in solution [32,33,34,35,36,37,38,39,40,41,42,43,44,45,46,47,48,49,50,51,52,53,54]. Results show that the photodecomposition of tetrazoles always involves cleavage of the tetrazolyl ring, leading to a variety of photoproducts. For these compounds, the structure of the substituents present in the tetrazole ring was found to strongly determine the nature of the photoproducts. Two other factors that contribute to the diversity in the photodegradation pathways and the variety of photoproducts are the possibility of tautomerism (which is associated with the presence in the molecule of labile hydrogen atoms) and the conformational flexibility of the substituents. In the case of substituted tetrazoles, tautomerism may also involve substituent groups [32,33,34]. In general, the presence of labile hydrogen atoms (either directly linked to the tetrazole ring or belonging to the tetrazole substituents) is a source of complexity in photochemical reactions that opens additional reaction channels or allows for secondary photochemical reactions to take place concomitantly with the main primary photoprocesses [32,33,34,41,42,43]. When substituents are linked to the tetrazole ring, the photochemistry of the molecule can also be influenced by the their conformational flexibility, which may favour or exclude certain reaction channels [35,36,37,38,39,40], determining the precise nature and relative amount of the final photoproducts.

This diversity in photodegradation pathways and putative photoproducts has rendered tetrazoles a permanent challenge to photochemists. If the photochemistry of tetrazoles is considered within a frame of potential development of synthetic methodologies the task is even more challenging, since the ultimate goal is to achieve selectivity. This may be difficult, because photochemical processes often lead to mixtures of primary and secondary photoproducts. 

**Figure 2:**
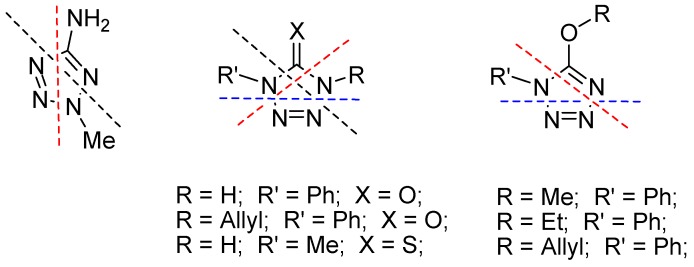
Photodecomposition patterns observed for several tetrazole derivatives in criogenic matrices.

The photochemistry of tetrazoles isolated in cryogenic inert matrices, has recently been reviewed [45,46]. Matrix isolation coupled to a suitable spectroscopic technique, such as FTIR, provides an excellent approach for the investigation of the photodegradation pathways, enabling the detection and charaterisation of unstable intermediates. For the range of compounds investigated, various fragmentation patterns were established (Figure 2), involving photocleavage of the formally single bonds on the heterocycle, and several photoproducts were produced on the matrix, often leading to the identification and characterization of new species [32,33,34,35,38,39,40]. However, their isolation and extraction is difficult, and therefore these processes are not easily applicable to organic synthesis.

The photochemistry of tetrazoles in solution is a more versatile strategy for synthetic applications, since a careful choice of solvent may lead to selectivity in photodegradation pathways and increase the stability of photoproducts through solvation. An important contribution to the available data on the photochemistry of tetrazole derivatives in solution is due to the work of Quast and co-workers during the 1980s [42,43,47,48,49,50,52]. Since then, other important contributions were added to the field, and some valuable information regarding applications of the photochemistry of tetrazoles in the development of synthetic methodologies is available in the literature. Also, recent experiments conducted in our labs indicate that cellulose induces selectivity in the photocleavage of some tetrazole derivatives. 

In the past decade, new effective synthetic routes to tetrazoles were published. Of special importance in the present context is the work by Sharpless and co-workers [55] on the development of efficient synthetic route to 5-substituted tetrazoles, by addition of azide to organic nitriles, catalyzed by zinc salts, in water. This review provides a summary of the photochemical transformations of tetrazole derivatives that lead to the formation of thermodynamically stable molecules, which, as such, can be used as target compounds or as building blocks in the development of new synthetic processes.

## 2. Photolysis of Tetrazoles as a Synthetic Tool for Easy Access to New Compounds 

Most of the contributions to the available knowledge on the photochemistry of tetrazole-based compounds reported in the last three decades were directed to the investigation of the photodegradation mechanisms of these nitrogen heterocycles. Moreover, the number of publications describing synthetically valuable photochemical processes by photolysis of tetrazoles is limited, since the number of compounds likely to be isolated from the reaction medium, or trapped, after irradiation, is not particularly large. This article reviews the photochemical transformations of tetrazoles that can be used in synthetic strategies, either for the production of final targets or the preparation of intermediate compounds. Each section relates to the preparation of a particular class of compounds, by direct photolysis of tetrazole derivatives.

### 2.1. 9H-Pyrimido[4,5-b]indoles from photolysis of biaryltetrazoles 

**Scheme 2:**
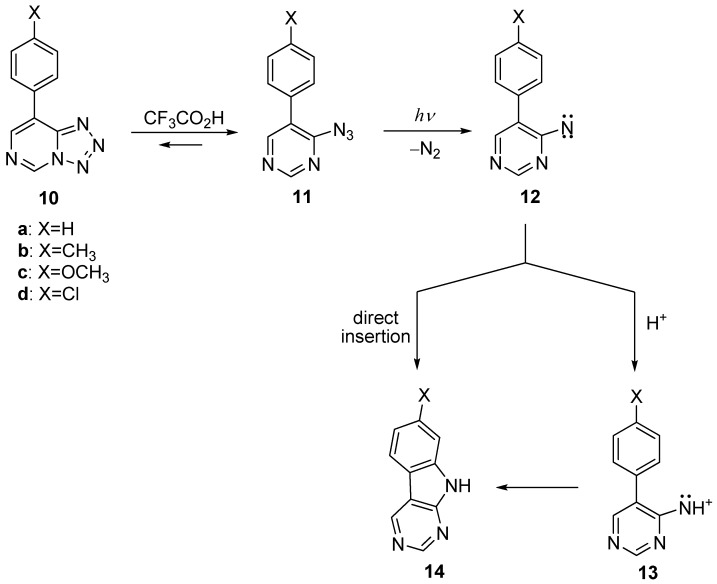
Photolysis of 8-(*p*-substituted)tetrazolo[1,5-*c*]-pyrimidines **10a-d** in trifluoro-acetic acid.

In 1972, Swenton *et al*. demonstrated that photolysis of biaryltetrazoles **10a-d** in acidic media leads to 9*H*-pyrimido[4,5-*b*]indoles **14a-d** in good to excellent yields (84–95%) ( Scheme 2) [51]. The authors presented a mechanistic proposal, according to which the reaction involves a shift of the tetrazole-azidoazomethine equilibration in acid media to the azide tautomer and its subsequent photolysis (16 RPR-3000-Å lamp). The initial photochemical reaction involves photoextrusion of molecular nitrogen, producing the corresponding nitrene **12**, followed by nitrene insertion to produce **14**. However, in the highly acidic trifluoroacetic acid, protonation would result in formation of the nitrenium ion **13**, which would be most reasonably followed by cyclization and proton loss to yield **14**. The authors demonstrated that substituent effects on the photolysis are negligible for the range of compounds studied, and chemical diversity may therefore be introduced.

### 2.2. Diaziridinones from photolysis of 1,4-dialkyl-1,4-dihydro-5H-tetrazol-5-ones and 1-alkenyl-4-alkyl-1,4-dihydro-5H-tetrazol-5-ones

**Scheme 3:**
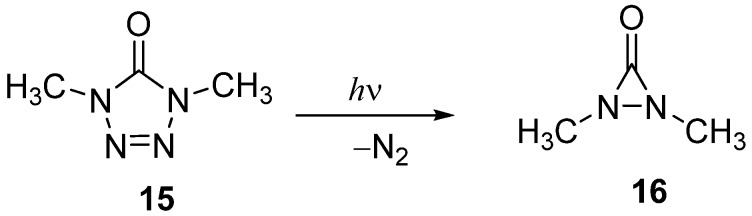
Synthesis of diaziridinone **16** from photolysis of tetrazolone **15**.

In 1975 Quast and Bieber described the photochemical synthesis of diaziridinone **16** from photolysis of 1,4-dimethyl-1,4-dihydro-5*H*-tetrazol-5-one (**15**) (Scheme 3) [43]. In the course of their investigation, the authors observed that photolysis of the thermally highly stable tetrazolone **15** in ether or 2-propanol afforded only secondary products from radical reactions of primary photoproducts with the solvent. However, when the irradiation of **15** (degassed samples at 10^−5^ Torr, 150 or 450 W Hg lamp) was conducted in CD_3_CN a single photoproduct was formed, characterized by ^1^H-NMR and IR as 1,2-dimethyldiaziridinone (**16**). 

**Scheme 4:**
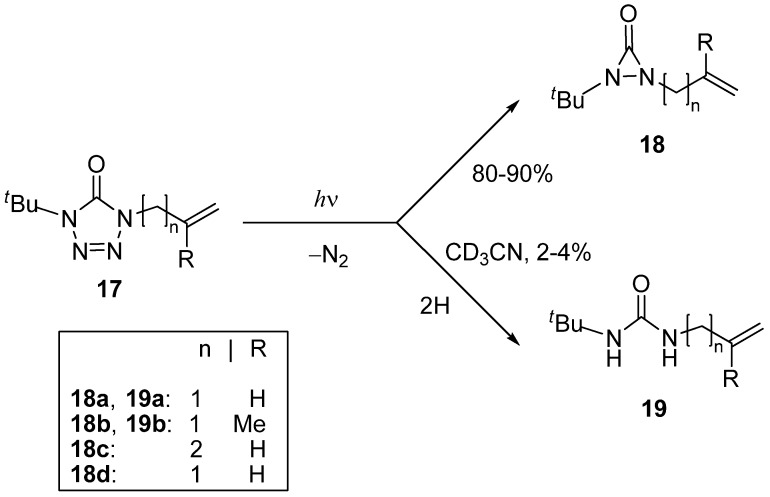
Synthesis of alkenyl-diaziridinones **18** from photolysis of tetrazolones **17**.

Eight years later, Quast and Nahr reported the formation of 1-alkenyl-2-alkyl-diaziridinones **18** via photoextrusion of nitrogen from 1-alkenyl-4-alkyl-1,4-dihydro-5*H*-tetrazol-5-ones **17a-d** [52]. Photolysis of tetrazolones **17** (254 nm, 20 °C, degassed samples at 10^−5^ Torr., 150 W Hg lamp) was conducted in [D_3_]acetonitrile, [D_12_]cyclohexane and [D_14_]methylcyclohexane, and diaziridinones **18a-d** were formed in 80–90% yield (Scheme 4), together with small amounts (<10%) of byproducts. When photolysis was performed in [D_3_]acetonitrile, the by-product was identified as *N*-alkenyl-*N*-alkyl urea (**19**), possibly resulting from hydrolysis of the primary photoproduct **18**, or from its biradical precursor.

### 2.3. Benzimidazolones from 1,4-dihydro-1-phenyl-5H-tetrazol-5-ones

**Scheme 5:**
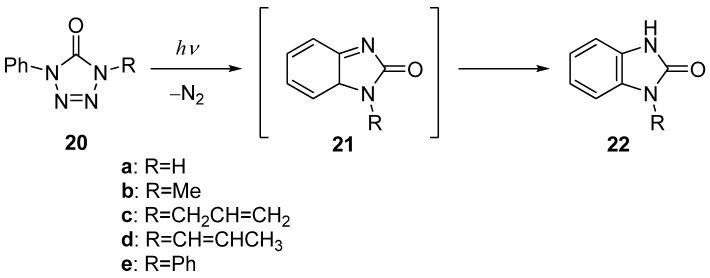
Synthesis of benzimidazolones **22** from photolysis of tetrazolones **20**.

In 1985, the same researchers reported the synthesis of benzimidazolones through photolysis of 1,4-dihydro-1-phenyl-5*H*-tetrazol-5-ones [49]. In this work, the authors irradiated a series of five (*N*-4)substituted 1-phenyl-tetrazolones **20a-e** (254 nm, 15 W Hg lamp, 20 °C) in methanol, acetonitrile or 2-propanol and observed photoextrusion of molecular nitrogen leading to benzimidazolones **22a-e** as final products in nearly quantitative yields. Proposed pathway involves formation of an intermediate that subsequently cyclises to **21** (Scheme 5), then rearranges to give **22**.

We have studied recently the UV-induced photochemistry (λ ≥ 235 nm) of 1-phenyltetrazolone **20a** isolated in solid argon [33]. Under these conditions, compound **20a** undergoes three different fragmentation pathways: *(i)* photo-induced [3+2] pericyclic molecular nitrogen elimination to give phenyldiaziridinone, which subsequently decomposes to afford isocyanic acid and cycloheptatetraene, *(ii)* photocleavage of the C(5)-N(1) and N(3)-N(4) bonds to yield phenylazide and isocyanic acid and *(iii)* photocleavage of the N(1)-N(2) and N(49-C(5) bonds to give phenylisocyanate and azide. Thus, formation of **22a** was not observed in solid argon.

We have also investigated the uv-induced photochemistry (λ ≥ 235 nm) of 1-phenyl-4-allyl-tetrazolone **20c** isolated in solid argon [38]. Under these conditions, compound **20c**, undergoes three different fragmentation pathways: *(i)* photo-induced [3+2] pericyclic molecular nitrogen elimination to give 1-allyl-2-phenyldiaziridinone, which is subsequently converted in 1-allyl-1*H*-benzoimidazol-2(3*H*)-one **22c**, *(ii)* photocleavage of the C(5)-N(1) and N(3)-N(4) bonds to yield phenylazide and allylisocyanate and *(iii)* photocleavage of the N(1)-N(2) and N(49-C(5) bonds to give phenylisocyanate and allylazide. Thus, formation of **22c** was also observed in solid argon, but the process was not selective.

From the above, it appears that, unlike in solid argon, UV-induced photolysis of a range of tetrazolones in solution may occur through a sole photodegradation mechanism, if experimental conditions are carefully adjusted, affording diaziridinones or benzimidazolones, depending on the nature of the starting tetrazolones. Thus, the process is selective, and is therefore of synthetic utility.

### 2.4. Carbodiimides from 1,4-disubstituted-1,4-dihydro-1-phenyl-5H-tetrazol-5-thiones

In 1983, Quast and Nahr reported that photolysis of 1-allyl-4-alkyltetrazol-5-thiones **23** affords the corresponding carbodiimides **24** by photochemically induced extrusion of molecular nitrogen and sulfur [52]. The authors irradiated diluted degassed solutions of 1-allyl-4-alkyltetrazol-5-thiones (**23**; R = allyl; R’ = alkyl) in [D_14_]-methylcyclohexane and [D_3_]-acetonitrile (λ ≥ 254 nm; high pressure mercury lamp; 20 °C) and the yields of carbodiimide obtained ranged from 50 to 80% yield. Two years later, in another publication, the same researchers reported formation of carbodiimides by photolysis of 4-alkenyl-, allyl-, or vinyl-1-phenyltetrazol-5-thiones **23** [49]. Solvents used were [D_3_]-acetonitrile, [D_4_]-methanol and dichloromethane, and irradiation conditions were those described above (λ ≥ 254nm; high pressure mercury lamp; 20 °C). Reported yields of conversion are above 80%. When irradiations were carried out in acetonitrile, the carbodiimide was converted to the corresponding urea.

We studied the photochemistry of 5-mercapto-1-methyltetrazole in solid argon [34]. In this environment, photolysis leads to formation of *(i)* 1-methyl-1-*H*-diazirene-3-thiol through photo-induced elimination of molecular nitrogen, *(ii)* methylisothiocyanate and azide, through photo-induced cleavage of bonds N(1)-N(2) and N(4)-C(5), *(iii)* N-methylcarbodiimide, through simultaneous extrusion of molecular nitrogen and sulfur. Again, photochemistry of 1,4-disubstituted-1,4-dihydro-5*H*-tetrazol-5-thiones in solution appears to be more selective than in cryogenic matrices, occurring exclusively through pathway (iii).

### 2.5. Iminoaziridines and hexahydronaphthyridines from photolysis of alkylidenedihydrotetrazoles 

**Scheme 7:**
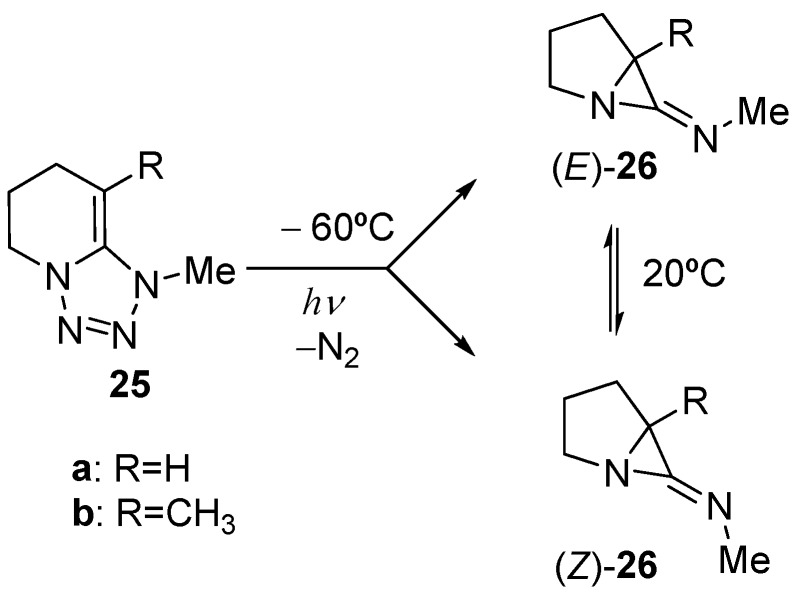
Synthesis of iminoaziridines **26a-b** by photolysis of alkylidenedihydrotetrazoles **25a-b**.

**Scheme 8:**
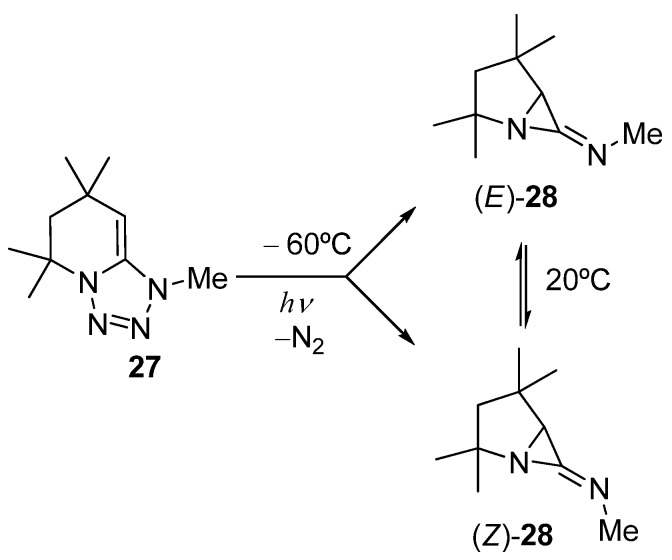
Synthesis of iminoaziridine **28** by photolysis of alkylidenedihydrotetrazole **27**.

**Scheme 9:**
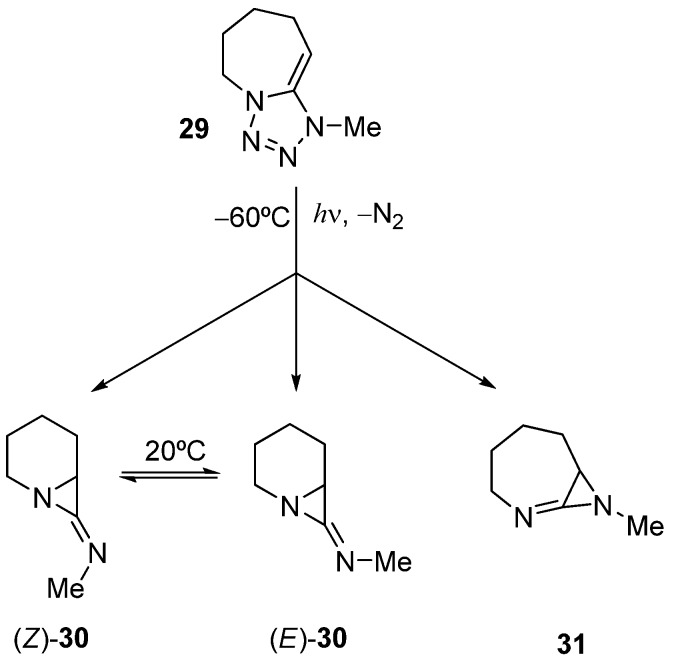
Synthesis of iminoaziridines **30** and **31** by photolysis of alkylidene-dihydrotetrazole **29**.

**Scheme 10:**
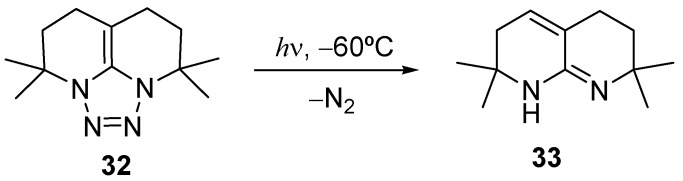
Synthesis of hexahydronaphthyridine **33** from photolysis of alkylidenedihydrotetrazole **32b**.

In 1998, Quast and Fuss described the synthesis of different annulated iminoaziridines by photolysis of 5-alkylidene-4,5-dihydro-1*H*-tetrazoles in solution [53]. During this study, diluted degassed solutions of alkylidenedihydrotetrazoles **25**, **27** and **29** were irradiated (λ ≥ 305 or 320 nm; high pressure mercury lamp) in [D_8_]toluene, affording annulated-iminoaziridines with an exocyclic CN double bond, *i.e.* derivatives **26**, **28**, and **30**, respectively (Scheme 7, Scheme 8 and Scheme 9) through extrusion of molecular nitrogen. Also, an equal amount of the isomer **31** with the endocyclic CN double bond is reported from **29**. In addition, irradiation (λ ≥ 320 nm) of a solution of alkylidenedihydrotetrazole **32b** in [D_8_]toluene at −60 °C afforded the hexahydronaphthyridine **33** quantitatively (Scheme 10). In a number of experiments, low temperature (−60 °C) was maintained during irradiation and recording of ^1^H-NMR spectra, precluding thermal (*E*) ↔ (*Z*) equilibration of the photoproducts **25**, **27** and **29**. Declining of the original yellow colour during irradiation indicated the disappearance of the alkylidenedihydrotetrazoles, which occurred slower at −60 °C than at 20 °C. Besides, the photolysis was monitored by NMR spectroscopy, which provided the evidence to the photoproduct structures. Some of the alkylidenedihydrotetrazoles investigated in this work were also irradiated in [D_6_]benzene and [D_6_]tetrahydrofuran. In all cases except for **25a**, the conversion and the yields, determined by comparison with the internal standard *t*-butyl methyl ether, were higher than 95%, and no by-products were identified. Just derivative **25a** formed considerable amounts of unidentified by-products, and, therefore, the yield of **26a** dropped to 30–50% (see Table 1).

### 2.6. 3,4-Dihydro-6-substituted-3-phenylpyrimidin-2(1H)-ones from 1,4-dihydro-1-allyl-4-phenyl-5H-tetrazol-5-ones

**Scheme 11:**
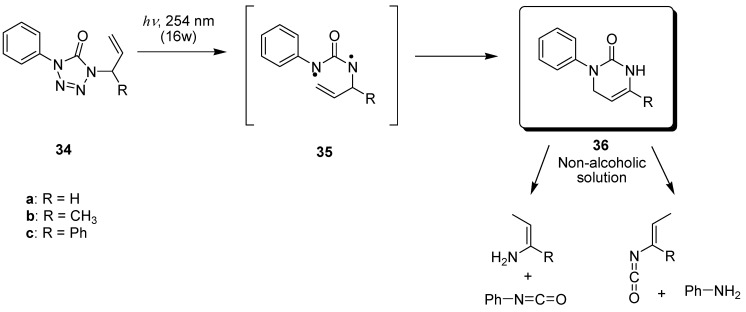
Synthesis of 3,4-dihydro-6-substituted-3-phenylpyrimidin-2(1*H*)-ones **36a-c** from photolysis of 4-allyl-tetrazolones **34a-c**.

The photochemistry of 1-allyl-4-phenyltetrazolones **34a-c** in solution was recently investigated by Cristiano and co-workers [36,37]. Solutions of compounds **34a-c** were irradiated (λ = 254 nm, 16 W low-pressure Hg lamp, 25 °C) in cyclohexane, acetonitrile, methanol, 1-propanol and 1-hexanol (Scheme 11). Gas evolution from the solution was observed, corresponding to the photoeliminated molecular nitrogen. Photolysis of compounds **34a-c** in cyclohexane, carbon tetrachloride and acetonitrile, resulted in formation of 3,4-dihydro-6-substituted-3-phenylpyrimidin-2(1*H*)-ones **36a-c** as sole primary photoproducts. However, in these solvents, the photoproducts **36a-c** were photochemically unstable, undergoing a rapid decomposition to afford a mixture of products identified as allyl amine and aniline (resulting from the secondary photoproducts allyl- and phenyl-azide), phenyl-, and allyl-isocyanates (Scheme 11). Photolysis of the same tetrazolones **34a-c** in the protic solvents methanol, 1-propanol or 1-hexanol also led to formation of 3,4-dihydro-6-substituted-3-phenylpyrimidin-2(1*H*)-ones **36a-c** as the sole primary photoproducts. However, in these solvents, pyrimidinones **36a-c** remained photostable even after extended periods of irradiation, and no secondary photoproducts were ever detected throughout the exposure. The products are formed in nearly quantitative yields and their isolation is carried out by simple evaporation of the solvent under reduced pressure, in mild conditions.

**Figure 3:**
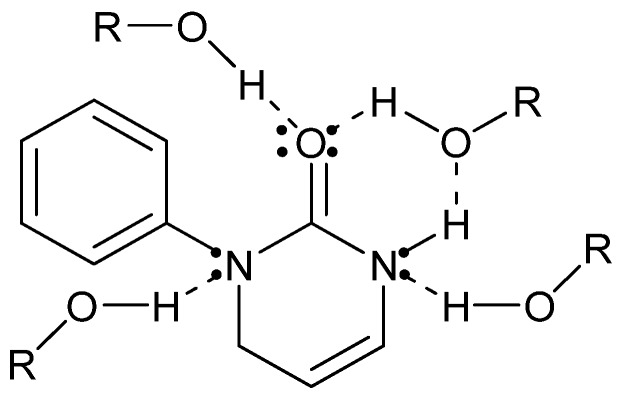
Stabilisation of pyrimidinone **36a** through solvation, in alcoholic solutions.

Mechanistically, we postulated that photoexcitation of 4-allyl-tetrazolones **34** leads to elimination of molecular nitrogen, yielding the triplet biradical intermediate **35**. In a second step, this intermediate rapidly undergoes ring closure and a 1,2-migration of hydrogen to form the pyrimidinone **36**, in an exothermic process. This mechanistic proposal was supported by the effect of the solvent viscosity on the photolysis quantum yields, and the sensitizing effect of the dissolved oxygen upon the photodegradation of tetrazolone, interpreted as a consequence of the T→S conversion of triplet biradicals, opening the way to the formation of the product **36**. Both effects were explained by the involvement of a caged triplet radical pair. The photostability exhibited by the pyrimidinones in alcoholic solutions, as opposed to cyclohexane, acetonitrile and carbon tetrachloride, is due to an efficient solvation through strong association with solvent molecules. Pyrimidinones bear several putative atoms capable of forming hydrogen bonds with solvent molecules, as depicted in Figure 3. Product solvation would then be very efficient, through stable ‘cages’ enclosing the pyrimidinone molecules and preventing their photodecomposition. The influence of these *cage effects* is also related to the kinetic energy of the primary photoproducts and to the viscosity of the solvent, affecting the photolysis quantum yields. In the alcoholic solutions, the pyrimidinones have more difficulty in forming molecular fragments upon excitation due to the ‘solvent cage’, this effect increasing with the increase in alcohol viscosity. Also, the absorbed energy is more efficiently dissipated though the solvated complex. The introduction of substituents on the allylic part (e.g. phenyl in **34c**) only affects the time required for complete conversion (around 5 hours for **34c**, as opposed to 3 hours for compound **34a**). Thus, we consider that the methodology can be applied to a wider range of 4-allyl-1-substituted tetrazolones, allowing for the introduction of chemical diversity. This methodology provides a valid access to a range of 3,4-dihydro-6-substituted-3-phenylpyrimidin-2(1*H*)-ones that may be stored as stable compounds after isolation.

Considering the relevance of this type of compounds in agriculture, we are now investigating the photochemistry of 1-allyl-4-aryl tetrazolones in cellulose. Results have shown that, upon irradiation (λ = 254 nm, 16 W low-pressure Hg lamp, 25 °C), compounds **34a-c** undergo complete conversion into the corresponding pyrimidinones **36a-c** in 1-2 minutes. Thus, the photocleavage in cellulose is selective and high yielding, affords the same photoproducts as in solution, but occurs at a much higher rate. Research is ongoing with the aim of understanding the basis of the catalytic effect exhibited by the cellulose matrix. 

### 2.7. Oxazines from 5-allyloxy-1-aryl-1H-tetrazoles

**Scheme 12:**
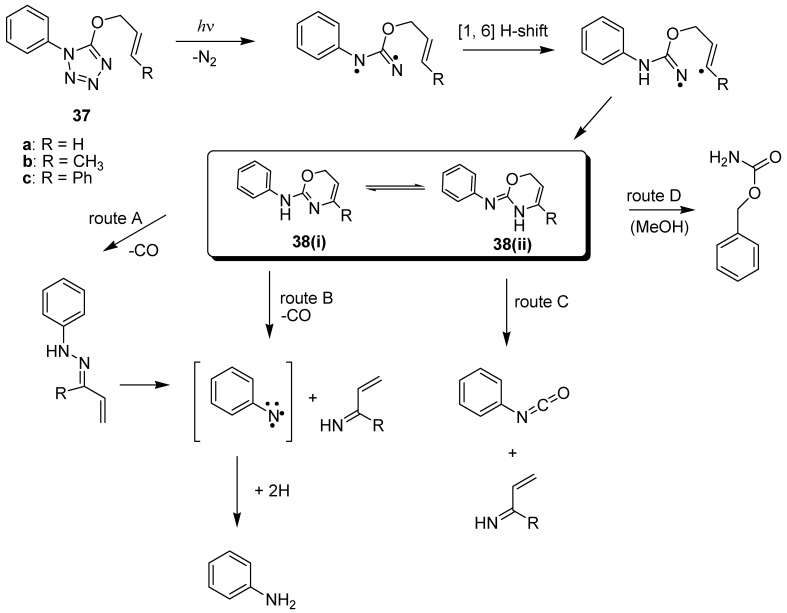
Synthesis of *N*-phenyl-1,3-oxazines **38a-c** from photolysis of 5-allyloxy-1-aryl-tetrazoles **37a-c**.

The photochemistry of 5-allyloxy-1-aryl-tetrazoles was also investigated by Cristiano and co-workers [40]. Compounds **37a-c** were irradiated with a low-pressure mercury lamp (λ = 254 nm), in methanol, acetonitrile and cyclohexane. Photolysis of ethers **37a-c** led to formation of *N*-phenyl-1,3-oxazines **38a-c** as the sole primary photoproduct (Scheme 12), resulting from photoextrusion of molecular nitrogen. However, oxazines **38a-c** could only be recovered in around 30% yield, due to their low photostability in solution. Chromatographic analysis revealed that, after a conversion of compounds **37a-c** of around 30%, secondary photoproducts resulting from photodecomposition of oxazines **38a-c** start to be detected (Scheme 12). Thus, oxazines may be isolated, but the yield of recovery from photolysed solutions is relatively low.

Oxazines **38a-c** can adopt two tautomeric forms, depending on the position of the amino function on the molecule. The NH group can act as a bridge, connecting the oxazine and phenyl rings, or this group can be alternatively included in the oxazine ring. DFT calculations performed at the B3LYP/6-31G(d,p) level of theory were carried out for all conformers of the two tautomeric forms of oxazines, **38a** and **38b**. The results obtained led to the identification of two low-energy local minima when the NH group is connected to the two rings [structure **38**(**i**)], and of three low-energy local minima when the NH function is included in the oxazine ring [structure **38**(**ii**)]. Because of the significant energy differences between the tautomers **38**(**i**) and **38**(**ii**), it can be expected that the population of oxazines at 25 °C will be dominated by structures **38**(**i**). Indeed, for all compounds **38a-c**, predicted populations of forms **38**(**i**) exceed 96%. The contribution of the minor tautomer **38**(**ii**) to the equilibrium mixture was predicted to range from about 2 to 4%, for isolated molecules in vacuum. It is important to call attention to the fact that the total dipole moments of tautomers **38**(**ii**) are systematically higher than those of the tautomers **38**(**i**). Thus, it may be expected that, in polar media, forms **38**(**ii**) will undergo additional stabilization with respect to forms **38**(**i**), and the relative population of the minor conformer will increase. Thus, both the tautomers **38**(**i**) and **38**(**ii**) are relevant for further photolysis of oxazines **38a-c** in solution, and the secondary photoproducts of 5-allyloxy-tetrazoles **37a-c** will be formed *via* photodecomposition of forms **38**(**i**) and **38**(**ii**), as presented in Scheme 12.

Considering the importance of oxazines, and the interest in developing synthetic methodologies for their easy preparation, we are now investigating the effect of other reaction media on the photolysis of 5-allyloxy-1-aryl-tetrazoles and on the stabilization of their primary photoproducts. Specifically, we are exploring the influence of matrices such as cellulose or silica on the photolysis and stability of photoproducts.

We recently investigated the photochemistry of 5-alkoxy-1-phenyltetrazoles isolated in solid argon [35,39]. In this media, photolysis leads to the formation of alkylcyanate and phenylazide, resulting from photoinduced cleavage of the C(5)-N(1) and N(3)-N(4) bonds, as the major photodegradation pathway. Another photofragmentation channel also observed results from cleavage of N(1)-N(2) and N(3)-N(4) bonds, leading to photo-elimination of molecular nitrogen and formation of 3-ethoxy-1-phenyl-1*H*-diazirene.

### 2.8. Iminodiaziridines from 5-imino-4,5-dihydro-1H-tetrazoles

The photochemistry of 5-imino-4,5-dihydro-1*H*-tetrazoles **39** was recently studied [54]. The authors irradiated diluted degassed solutions of 5-imino-4,5-dihydro-1*H*-tetrazoles in [D_8_]-tetrahydrofuran (λ ≥ 254 nm; high pressure mercury lamp; −60 °C). The irradiation was conducted in sealed NMR tubes and the reaction monitored by NMR. Results indicated the intervention of a major photofragmentation channel leading to elimination of molecular nitrogen and formation of iminoaziridines **40-42**, in yields above 80%, and a minor photofragmentation channel leading to *N,N*-dialkylcarbodiimide and alkyl azide. Although this procedure leads to a mixture of isomers, and there are two fragmentation pathways involved, this work is very relevant in mechanistic terms, because it brings further evidence for the involvement of singlet biradicals in photodegradation pathways of tetrazoles. The authors proposed the formation of triaza trimethylene methane diradicals as the intermediate species resulting from initial photo induced extrusion of molecular nitrogen. Biradical intermediates had already been proposed by us for the photofragmentation of 5-allyloxy-1-phenyltetrazoles and 1,4-dihydro-1-allyl-4-phenyltetrazol-5-ones in solution [36,37,40].

## 3. Conclusions

Photolysis of tetrazole derivatives in solution may be an attractive synthetic methodology for the preparation of other compounds. Through a careful selection of solvent and other reaction conditions, the photofragmentation process may be tuned to grant selectivity, affording stable and synthetically useful photoproducts that may be isolated and stored, or trapped in the reaction media. A diversity of photoproducts such as 9*H*-pyrimido(4,5-b) indoles, diaziridinones, iminoaziridines, iminodiaziridines, carbodiimides, benzimidazolones, pyrimidinones and oxazines may be trapped, or isolated and used. It is interesting to note that, for all tetrazoles studied, the photodegration pathway in solution always leads to extrusion of molecular nitrogen. This contrasts with photochemistry in cryogenic matrices, where a plethora of pathways and photoproducts may be at play. Preliminary results for the photolysis of tetrazoles in cellulose matrices indicate that this media may be important in the development of efficient and selective synthetic strategies based on the photolysis of tetrazoles.

## Figures and Tables

**Scheme 6:**
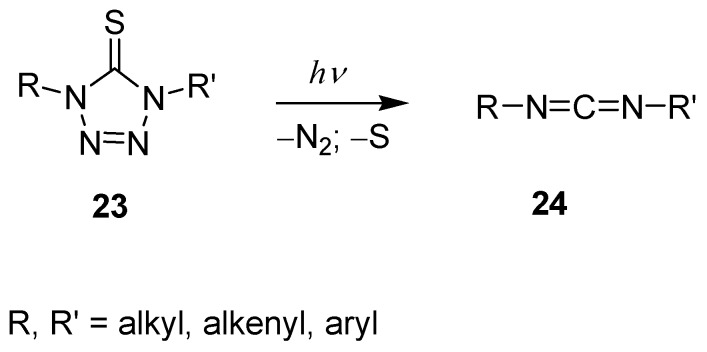
Synthesis of carbodiimides **24** from 1,4-disubstituted-1,4-dihydro-1-phenyl-5*H*-tetrazol-5-thiones **23**.

**Scheme 13:**
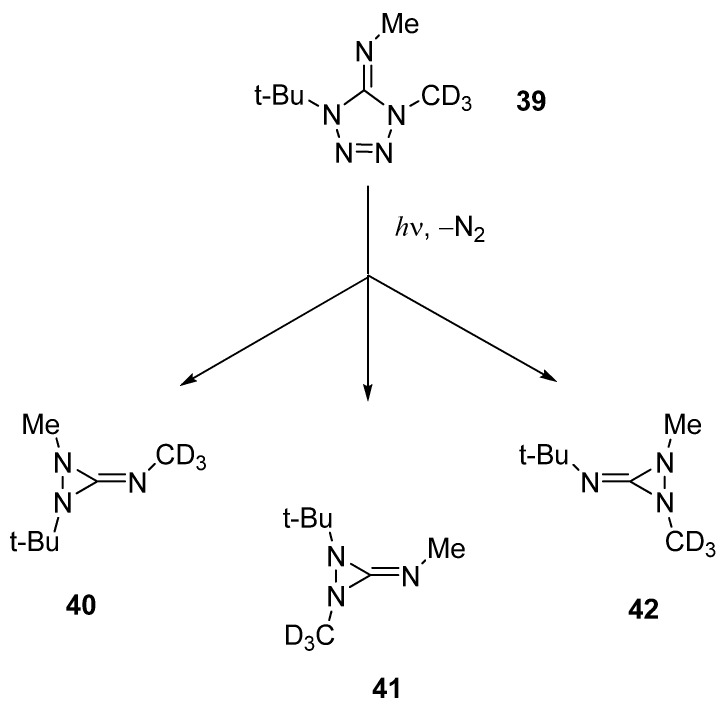
Synthesis of iminodiaziridines **40-42** from photolysis of 5-imino-4,5-dihydro-1*H*-tetrazoles **39**.

**Table 1:** Experimental conditions and results for the photolysis of alkylidenedihydrotetrazoles **25**, **27**, **29** and **32**. The conversions and the yields were higher than 95%, except for the cases referred on the footnotes.

Compound	λ (nm)	Temp. (°C)	Time (h)	Product	Solvent^(a)^
**25a**	≥ 320 ≥ 305	−60 20	3.5 0.75	**26a**^(b)^ ^(c)^	T B
**25b**	≥ 320	−60	4	**26b**	T
**27**	≥ 320 ≥ 320 ≥ 320	−60 20 20	3 0.67 0.33	**28**	T A B
**29**	≥ 320 ≥ 305	−60 20	3.5 3	**30:31** (47:53) (49:51)	T B
**32**	≥ 320	−60	1.25	**33**	T

^(a)^ Solvent A: [D_8_]tetrahydrofuran, B: [D_6_]benzene, T: [D_8_]toluene; ^(b)^ 30% yield; ^(c)^ 50% yield.

## References

[1] Bugalho S.C.S., Maçoas E.M.S., Cristiano M.L.S., Fausto R. (2001). Low temperature matrix-isolation and solid state vibrational spectra of tetrazole. Phys. Chem. Chem. Phys..

[2] Butler R.N. (1996). Comprehensive Heterocyclic Chemistry, II.

[3] Butler R.N. (1984). Comprehensive Heterocyclic Chemistry, I.

[4] Catalan J., Abboud J.L.M., Elguero J. (1987). Basicity and acidity of azoles. Adv. Het. Chem..

[5] Elguero J., Marzin C., Katritzky A.R., Linda P. (1976). The Tautomerism of Heterocycles.

[6] Brigas A.F., Storr R.C., Gilchrist T.L. (2004). Tetrazoles. Science of Synthesis, Houben-Weyl Methods of Molecular Transformations.

[7] Koldobskii G.I., Ostrovskii V.A. (1988). Acid-base properties of 5-membered nitrogen-heterocycles. Khim. Get. Soed..

[8] Koldobskii G.I., Ostrovskii V.A. (1994). Tetrazoles. Uspek. Khim..

[9] Koldobskii G.I., Ostrovskii V.A., Gidaspov B.V. (1980). Tautomerism and acid-base properties of tetrazoles - review. Khim. Get. Soed..

[10] Koldobskii G.I., Ostrovskii V.A., Poplavskii V.S. (1981). Advances in tetrazole chemistry. Khim. Get. Soed..

[11] Ostrovskii V.A., Koren A.O. (2000). Alkylation and related electrophilic reactions at endocyclic nitrogen atoms in the chemistry of tetrazoles. Heterocycles.

[12] Katritzky A.R., Pozharskii A.F. (2000). Handbook of Heterocyclic Chemistry.

[13] Katritzky A.R., Karelson M., Harris P.A. (1991). Prototropic tautomerism of heteroaromatic-compounds. Heterocycles.

[14] Noda K., Saad Y., Kinoshita A., Boyle T.P., Graham R.M., Husain A., Karnik S.S. (1995). Tetrazole and carboxylate groups of angiotensin receptor antagonists bind to the same subsite by different mechanisms. J. Bio. Chem..

[15] Mavromoustakos T., Kolocouris A., Zervou M., Roumelioti P., Matsoukas J., Weisemann R. (1999). An effort to understand the molecular basis of hypertension through the study of conformational analysis of Losartan and Sarmesin using a combination of nuclear magnetic resonance spectroscopy and theoretical calculations. J. Med. Chem..

[16] Toney J.H., Fitzgerald P.M.D., Grover-Sharma N., Olson S.H., May W.J., Sundelof J.G., Vanderwall D.E., Cleary K.A., Grant S.K., Wu J.K., Kozarich J.W., Pompliano D.L., Hammond G.G. (1998). Antibiotic sensitization using biphenyl tetrazoles as potent inhibitors of bacteroides fragilis metallo-beta-lactamase. Chem. Biol..

[17] Hashimoto Y., Ohashi R., Kurosawa Y., Minami K., Kaji H., Hayashida K., Narita H., Murata S. (1998). Pharmacologic profile of TA-606, a novel angiotensin II receptor antagonist in the rat. J. Card. Pharm..

[18] Desarro A., Ammendola D., Zappala M., Grasso S., Desarro G.B. (1995). Relationship between structure and convulsant properties of some beta-lactam antibiotics following intracerebroventricular microinjection in rats. Antimicrob. Agents Chemother..

[19] Abell A.D., Foulds G.J. (1997). Synthesis of a cis-conformationally restricted peptide bond isostere and its application to the inhibition of the HIV-1 protease. J. Chem. Soc. Perkin Trans. 1.

[20] Tamura Y., Watanabe F., Nakatani T., Yasui K., Fuji M., Komurasaki T., Tsuzuki H., Maekawa R., Yoshioka T., Kawada K., Sugita K., Ohtani M. (1998). Highly selective and orally active inhibitors of type IV collagenase (MMP-9 and MMP-2): N-sulfonylamino acid derivatives. J. Med. Chem..

[21] Sandmann G., Schneider C., Boger P. (1996). A new non-radioactive assay of phytoene desaturase to evaluate bleaching herbicides. Z. Naturf. C (Biosciences).

[22] Ostrovskii V.A., Pevzner M.S., Kofman T.P., Shcherbinin M.B., Tselinskii I.V. (1999). Targets in Heterocyclic Systems: Chemistry and Properties.

[23] Frija L.M.T., Fausto R., Loureiro R.M.S., Cristiano M.L.S. (2009). Synthesis and structure of novel benzisothiazole-tetrazolyl derivatives for potential application as nitrogen ligands. J. Mol. Cat. A Chem..

[24] Moore D.S., Robinson S.D. (1988). Catenated Nitrogen Ligands.2. Transition-metal derivatives of triazoles, tetrazoles, pentazoles, and hexazine. Adv. Inorg. Chem..

[25] Araujo N.C.P., Barroca P.M.M., Bickley J.F., Brigas A.F., Cristiano M.L.S., Johnstone R.A.W., Loureiro R.M.S., Pena P.C.A. (2002). Structural effects on sigmatropic shifts in heteroaromatic allyl ethers. J. Chem. Soc. Perkin Trans. 1.

[26] Araujo N.C.P., Brigas A.F., Cristiano M.L.S., Frija L.M.T., Guimaraes E.M.O., Loureiro R.M.S. (2004). Heteroaromatic benzyl ethers as intermediates for palladium-catalysed transfer hydrogenolysis of benzyl alcohols. J. Mol. Cat. A Chem..

[27] Barkley J.V., Cristiano M.L.S., Johnstone R.A.W., Loureiro R.M. S. (1997). 3-(E)-but-2-enoxy-1,2-benzisothiazole 1,1-dioxide: unusual C-O-C ether bond lengths and reactivity. Acta Crystallogr. C-Cryst. Struct. Commun..

[28] Cristiano M.L.S., Johnstone R.A.W. (1997). Intramolecularity of the thermal rearrangement of allyloxytetrazoles to n-allyltetrazolones. J. Chem. Res. (S).

[29] Cristiano M.L.S., Johnstone R.A.W. (1997). A kinetic investigation of the thermal rearrangement of allyloxytetrazoles to N-allyltetrazolones. J. Chem. Soc. Perkin Trans. 2.

[30] Frija L.M.T., Cristiano M.L.S., Guimaraes E.M.O., Martins N.C., Loureiro R.M.S., Bickley J.F. (2005). Palladium-catalysed reduction of heteroaromatic naphthyl ethers: Structural effects on reactivity. J. Mol. Cat. A Chem..

[31] Maier G., Eckwert J., Bothur A., Reisenauer H.P., Schmidt C. (1996). Photochemical fragmentation of unsubstituted tetrazole, 1,2,3-triazole, and 1,2,4-triazole: First matrix-spectroscopic identification of nitrilimine HCNNH. Lieb. Annal..

[32] Gómez-Zavaglia A., Reva I.D., Frija L., Cristiano M.L., Fausto R. (2005). Molecular structure, vibrational spectra and photochemistry of 2-methyl-2*H*-tetrazol-5-amine in solid argon. J. Phys. Chem. A.

[33] Gómez-Zavaglia A., Reva I.D., Frija L., Cristiano M.L., Fausto R. (2006). Photochemistry of 1-phenyl-tetrazolone isolated in solid argon. J. Photochem. Photobiol. A Chem..

[34] Gómez-Zavaglia A., Reva I.D., Frija L., Cristiano M.L., Fausto R. (2006). Molecular structure, vibrational spectra and photochemistry of 5-mercapto-1-methyltetrazole. J. Mol. Struct..

[35] Gómez-Zavaglia A., Reva I.D., Frija L., Cristiano M.L.S., Fausto R. (2006). Infrared spectrum and UV-induced photochemistry of matrix-isolated 5-methoxy-1-phenyl-1*H*-tetrazole. J. Photochem. Photobiol. A Chem..

[36] Frija L.M.T., Khmelinskii I.V., Cristiano M.L.S. (2005). Novel efficient synthesis of 3,4-dihydro-6-substituted-3-phenylpyrimidin-2-(1*H*)-ones. Tetrahedron Lett..

[37] Frija L.M.T., Khmelinskii I.V., Cristiano M.L.S. (2006). Mechanistic investigations into the photochemistry of 4-allyl-tetrazolones in solution: A new approach to the synthesis of 3,4-dihydro-pyrimidinones. J. Org. Chem..

[38] Frija L.M.T., Reva I.D., Gómez-Zavaglia A., Cristiano M.L.S., Fausto R. (2007). UV-induced photochemistry of matrix-isolated 1-phenyl-4-allyl-tetrazolone. Photochem. Photobiol. Sci..

[39] Frija L.M.T., Reva I.D., Gómez-Zavaglia A., Cristiano M.L.S., Fausto R. (2007). Photochemistry and vibrational spectra of matrix-isolated 5-ethoxy-1-phenyl-1*H*-tetrazole. J. Phys. Chem. A.

[40] Frija L.M.T., Khmelinskii I.V., Serpa C., Reva I.D., Fausto R., Cristiano M.L.S. (2008). Photochemistry of 5-allyloxy-tetrazoles: steady-state and laser flash photolysis study. Org. Biomol. Chem..

[41] Awadallah A., Kowski K., Rademacher P. (1997). Gas phase thermolysis of pyrazolines.5. Electronic structure and gas phase thermolysis of tetrazole derivatives studied by photoelectron spectroscopy. J. Het. Chem..

[42] Dunkin I.R., Shields C.J., Quast H. (1989). The photochemistry of 1,4-dihydro-5*H*-tetrazole derivatives isolated in low-temperature matrices. Tetrahedron.

[43] Quast H., Bieber L. (1975). Photochemical formation of methylenecyclopropane analogs.1. aziridinimines, diaziridinimines, diaziridinones, and carbodiimides by photolysis of 2-tetrazolines. Angew. Chem. Int. Ed. Engl..

[44] Dunkin I.R. (1986). The infrared-spectrum, light-induced infrared linear dichroism and conformational-analysis of phenyl azide in solid nitrogen at 12-K. Spectrochim. Acta Part A Mol. Biomol. Spectrosc..

[45] Gómez-Zavaglia A., Reva I.D., Frija L.M.T., Cristiano M.L.S., Fausto R. (2008). Photochemistry of Tetrazole Derivatives in Cryogenic Rare Gas Matrices. Photochemistry Research Progress.

[46] Gómez-Zavaglia A., Reva I.D., Frija L.M.T., Cristiano M.L.S., Fausto R. (2009). Photochemistry of tetrazole derivatives in cryogenic rare gas matrices. Chem. Phys. Res. J..

[47] Quast H., Bieber L. (1981). Photochemical-synthesis of methylencyclopropane analogs.5. Synthesis and photolysis of 1,4-dialkyl-1,4-dihydro-5*H*-tetrazol-5-ones and 1,4-dialkyl-1,4-dihydro-5*H*-tetrazol-5-thiones - a novel approach to diaziridinones and carbodiimides. Chem. Ber. Recl..

[48] Quast H., Fuss A., Nahr U. (1985). Photochemical formation of methylene cyclopropane analogs.11. photochemical elimination of molecular nitrogen from phenyl-substituted 1,4-dihydro-5-imino-5*H*-tetrazoles - products of phenyl substituted tris(imino)methane diradicals. Chem. Ber. Recl..

[49] Quast H., Nahr U. (1985). Photoextrusion of nitrogen from 1,4-dihydro-1-phenyl-5*H*-tetrazol-5-ones and 5-thiones - benzimidazolones and carbodiimides. Chem. Ber. Recl..

[50] Quast H. (1980). Some recent advances in the synthesis of 3-membered ring heterocycles. Heterocycles.

[51] Hyatt J.A., Swenton J.S. (1972). Photochemistry in the tetrazole-azidoazomethine system. A facile synthesis of 9*H*-pyrimido[4,5-b]indoles. J. Org. Chem..

[52] Quast H., Nahr U. (1983). Photchemische stickstoff-eliminierung aus 1-alkenyl-4-alkyl-1,4-dihydro-5*H*-tetrazol-5-onen und -thionen. Diaziridinone und carbodiimide mit alkenylsubstituenten. Chem. Ber. Recl..

[53] Quast H., Fuss A., Nüdling W. (1998). Photoextrusion of molecular nitrogen from annulated 5-alkylidene-4,5-dihydro-1*H*-tetrazoles: Annulated iminoaziridines and the first triplet diazatrimethylenemethane. Eur. J. Org. Chem..

[54] Quast H., Bieber L. (2008). Iminodiaziridines by regio- and stereoselective cyclization of diasterioisomeric singlet triazatrimethylenemethane diradicals generated through photolysis of 5-imino-4,5-dihydro-1*H*-tetrazoles. J. Org. Chem..

[55] Demko Z.P., Sharpless K.B. (2001). Preparation of 5-substituted 1*H*-tetrazoles from nitriles in water. J. Org. Chem..

